# *Acanthamoeba castellanii* of the T4 genotype is a potential environmental host for *Enterobacter aerogenes* and *Aeromonas hydrophila*

**DOI:** 10.1186/1756-3305-6-169

**Published:** 2013-06-07

**Authors:** Farzana Abubakar Yousuf, Ruqaiyyah Siddiqui, Naveed Ahmed Khan

**Affiliations:** 1 Department of Biological and Biomedical Sciences, Aga Khan University, Karachi, Pakistan

**Keywords:** *Acanthamoeba*, Interactions, Encystment, Vector, Pathogens

## Abstract

**Background:**

*Acanthamoeba* can interact with a wide range of microorganisms such as viruses, algae, yeasts, protists and bacteria including *Legionella pneumophila*, *Pseudomonas aeruginosa*, *Vibrio cholerae*, *Helicobacter pylori*, *Listeria monocytogenes*, *Mycobacterium* spp., and *Escherichia coli*. In this capacity, *Acanthamoeba* has been suggested as a vector in the transmission of bacterial pathogens to the susceptible hosts.

**Methods:**

Here, we used a keratitis isolate of *A. castellanii* of the T4 genotype and studied its interactions with two bacterial genera which have not been tested before, *Enterobacter aerogenes*, and *Aeromonas hydrophila*, as well as *E. coli*. Assays were performed to determine bacterial association with and invasion of *A. castellanii*. Additionally, bacterial survival intracellular of *A. castellanii* trophozoites as well as cysts was determined.

**Results:**

All three bacterial isolates tested, associated, invaded, and survived inside *A. castellanii* trophozoites as well as *A. castellanii* cysts. However, *E. aerogenes* and *E. coli* exhibited significantly reduced association with and invasion of *A. castellanii* as compared with *A. hydrophila* (*P* < 0.01 using paired T-test, one tail distribution). In the long term survival assays, all three bacterial isolates tested remained viable inside *A. castellanii* trophozoites, while amoeba remained intact; however *A. hydrophila* exhibited higher survival inside amoebae (14.54 ± 3.3 bacteria:amoeba ratio) compared with *E. aerogenes* (3.96 ± 0.7 bacteria:amoeba ratio) and *E. coli* (5.85 ± 1.1 bacteria:amoeba ratio). *A. hydrophila*, *E. coli*, and *E. aerogenes* remained viable during the encystment process and exhibited higher levels of recovery from mature cysts (14.13 ± 0.89 *A. hydrophila*:amoeba ratio, 10.13 ± 1.17 *E. aerogenes*:amoeba ratio, and 11.95 ± 0.7 *E. coli*:amoeba ratio).

**Conclusions:**

*A. hydrophila* and *E. aerogenes* also joined the ranks of other bacteria that could benefit from *A. castellanii*. Because cysts can be airborne, these findings suggest that *Acanthamoeba* is a potential vector in the transmission of *A. hydrophila* and *E. aerogenes* to susceptible hosts.

## Background

*Acanthamoeba* spp. were discovered in a culture of *Cryptococcus pararoseus* (fungus) by Castellani in 1930 [1-3]. Being free-living protists, *Acanthamoeba* spp. have the capability to endure harsh environments. There are two stages in the life cycle of *Acanthamoeba* i.e., a vegetative trophozoite stage and a resistant cyst stage. Bacteria, algae, yeasts or small organic particles are the source of nourishment for *Acanthamoeba* during the trophozoite stage, while harsh environmental conditions (i.e., lack of food, increased osmolarity or hypo-osmolarity, extremes in temperatures and pH) promote trophozoite conversion into the cyst stage. A number of studies have documented *Acanthamoeba* spp. as a reservoir for the survival of pathogenic bacteria in the environment [4], and suggested their potential role in the transmission of pathogenic microbes to the susceptible population, particularly in a clinical setting.

*Enterobacter aerogenes* (formerly known as *Klebsiella aerogenes*), a Gram negative bacterium belonging to the family of Enterobacteriaceae, is often associated with nosocomial infections [5]. The colonization of *E. aerogenes* most commonly occurs amongst intensive care unit (ICU) patients, usually in the respiratory, urinary, and gastrointestinal tracts and less frequently in skin and surgical wounds [6-10]. Another class of bacteria, i.e., *Aeromonas hydrophila* are Gram-negative enteropathogens that belong to the family Vibrionaceae. They are found in fresh water habitats throughout the world and infect cultured and feral fishes [11]. A number of epidemiological studies have demonstrated the presence of *A. hydrophila* in stools of young children with diarrhea. It has also been found to be associated with infections, such as endocarditis, gastroenteritis, hemolytic-uremic syndrome, meningitis, pneumonia, septicemia, urinary tract infections, wound infections, etc. [12]. In this study, we determined *A. castellanii* interactions with *E. aerogenes* and *A*. *hydrophila*.

## Methods

### Culture of acanthamoeba

All chemicals were purchased from Sigma Laboratories (St. Louis, USA) and Oxoid (Hampshire, England) unless otherwise stated. A clinical isolate of *A. castellanii* belonging to the T4 genotype, originally isolated from a keratitis patient (American Type Culture Collection, ATCC 50492) was used in the present study. *A. castellanii* was cultured in PYG medium containing [0.75% (w/v) proteose peptone, 0.75% (w/v) yeast extract and 1.5% (w/v) glucose)]. The amoebae were grown in tissue culture flasks at 30°C without shaking [13].

### Bacterial cultures

*E. aerogenes* (formerly known as *Klebsiella aerogenes*) is a clinical isolate available in the university microbial collection. A clinical isolate of *A. hydrophila* (diarrheal patient and kindly provided by Dr. Anita Zaidi, Aga Khan University Hospital) was used in the present study. In addition, *Escherichia coli* K1 strain E44, a spontaneous rifampin-resistant mutant of a CSF isolate of K1-encapsulated *E. coli* RS218 was used (O18:K1:H7) [14,15]. Bacteria were cultured in Luria Bertani (LB) overnight at 37°C without shaking prior to experimentation.

### Antibiotic sensitivity assays

For antibiotic susceptibility testing of *E. aerogenes* and *A. hydrophila*, the disk diffusion method was used according to Kirby–Bauer [16]. Tests were performed on Muller-Hinton agar and inhibition zone diameters were interpreted according to antimicrobial susceptibility CSLI guidelines [17].

### Association assays

*A. castellanii* were maintained in the trophozoite stage in tissue culture flasks in PYG medium. Upon confluency, the unbound amoebae were aspirated and growing trophozoites were washed once with phosphate buffered saline (PBS) pH 7.4. The growth medium, PYG (5 mL) was added to the flask and trophozoites were chilled on ice for 20 min, pelleted by centrifugation at 900× *g* for 5 min. The cell pellet was dispersed in 1 mL of PBS and the number of amoebae were counted using a haemocytometer (10^6^). For bacterial cultures, the optical density was adjusted to 0.22 at 595 nm [equivalent to approximately 10^8^ colony forming units (c.f.u.) per mL]. Bacteria (10^7^c.f.u.) were incubated with *A. castellanii* (10^6^ cells) at 30°C for 1 h. After the incubation, co-cultures of amoebae plus bacteria were transferred to a 1.5 mL eppendorf tube and centrifuged at 2000× *g* for 5 min. Next, the supernatant was aspirated and the pellet was resuspended in 0.5 mL of PBS and vortexed by a brief pulse. This process was repeated three times to remove non-associated bacteria. At the final wash, the discarded supernatant was also plated onto nutrient agar plates to determine bacterial presence. Finally, amoebae were counted using a haemocytometer. The amoebae were lysed by adding sodium dodecyl sulfate (SDS; 0.5% final concentration) and kept at room temperature for 10 min. The lysates containing bacteria were plated onto nutrient agar plates and colonies enumerated the next day [18]. The percentage of bacterial association was calculated as follows: recovered bacterial c.f.u. / total bacterial c.f.u. × 100 =% bacterial c.f.u. associated with *A. castellanii*. In addition, the ratio of bacteria to amoebae was calculated as follows: recovered bacterial c.f.u. / number of *A. castellanii* = bacterial c.f.u.:*A. castellanii* ratio.

### Invasion assays

The ability of bacteria to invade or be taken up by *A. castellanii* was observed by performing invasion assays. Briefly, the amoebae were grown to confluency in a trophozoite stage in tissue culture flasks followed by the addition of bacteria as described for association assays. *A. castellanii* were incubated for 1 h and washed three times with PBS. The extracellular bacteria were killed by adding gentamicin (100 μg per mL) and incubated for 45 min at 30°C. After incubation in gentamicin, amoebae were washed three times in 500 μL aliquots of PBS. At the final wash, the PBS was also plated onto nutrient agar plates to ensure any remaining extracellular bacteria had been killed. The washed amoebae were then resuspended in 500 μL of fresh PBS and enumerated using a haemocytometer. Finally, amoebae were lysed using 0.5% SDS together with vortexing. Aliquots of the lysate were plated on nutrient agar plates to determine the bacterial counts. The susceptibility of bacteria to gentamicin was tested independently by incubating cultures of bacteria grown overnight with 100 μg per mL as indicated above. The percentage of bacterial invasion/uptake was calculated as follows: recovered bacterial c.f.u. / total bacterial c.f.u. × 100 = % intracellular bacterial c.f.u.*.* In addition, the ratio of bacteria to amoebae was calculated as follows: recovered bacterial c.f.u./ number of *A. castellanii* = bacterial c.f.u.:*A. castellanii* ratio.

### Intracellular survival assays

The number of intracellular bacteria in *A. castellanii* was assessed by intracellular survival assays. Briefly, amoebae were incubated with bacteria, followed by the addition of gentamicin (100 μg per mL) for 45 min as for invasion assays. After this incubation, *A. castellanii* were washed three times with PBS and subsequently incubated in 0.5 mL of PBS for 24 h at 30°C. Finally, amoebae and bacterial c.f.u. were counted as described above and bacteria that had survived intracellularly were calculated as follows: recovered bacterial c.f.u. / total bacterial c.f.u. × 100 =% intracellular bacterial c.f.u. after 24 h in PBS. In addition, the ratio of bacteria to amoebae was calculated as follows: recovered bacterial c.f.u. / number of *A. castellanii* = bacterial c.f.u.:*A. castellanii* ratio.

### Intracellular cyst survival assays

Encystment assays were performed to evaluate the ability of bacteria to survive inside *A. castellanii* cysts. In brief, following invasion assays, the mixtures were transferred onto non-nutrient agar plates without any bacterial lawn [prepared using 3% (w/v) purified agar]. The plates were incubated at room temperature for up to 10 days. This allowed a complete encystment of *A. castellanii* trophozoites into the cyst form, as observed visually under a phase-contrast microscope. Cysts were then gently scraped-off the agar surface using a cell scraper by adding 5 mL of sterile deionized water and collected by centrifugation at 900× *g* for 10 min and resuspended in 0.5 mL of sterile deionized water and counted using a haemocytometer. The cysts were treated with SDS (0.5% final concentration) and the associated numbers of bacteria were enumerated by plating onto nutrient agar plates. The bacterial counts were calculated as follows: recovered bacterial c.f.u./ total bacterial c.f.u. × 100 =% bacterial c.f.u.. The ratio of bacteria to amoebae was calculated as follows: recovered bacterial c.f.u. / number of *A. castellanii* cysts = bacterial c.f.u.:*A. castellanii* cysts ratio. For controls, *A. castellanii* cysts were prepared in the absence of bacteria.

## Results

Antibiotic susceptibility testing revealed that both *E. aerogenes* and *A. hydrophila* were sensitive to amoxicillin/clavulanic acid, amikicin, aztreonam, imipenem, iperacillin,/tazobactam, gentamicin, ceftriaxon, cefuroxime, ofloxacin, sulphamethoxazole/trimethoprim, cefixime, chloramphenicol but resistant to ampicillin.

### *A. hydrophila* displayed higher association with and invasion/uptake by *A. castellanii* as compared to *E. coli* and *E. aerogenes*

**Figure 1:**
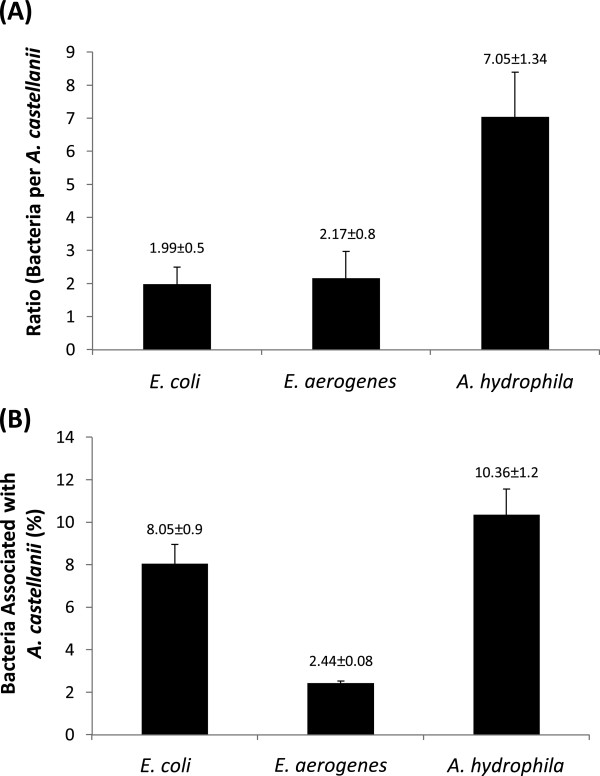
***Acanthamoeba castellanii *exhibited reduced association with *Enterobacter aerogenes *compared with *Aeromonas hydrophila*.** Association assays were performed to determine bacterial association with *A. castellanii* as described in “Methods”. (**A**) represents ratio of bacteria per amoeba, while (**B**) represents bacteria associated with amoeba (% of the original inoculum). Results are represented as the mean ± standard error of three independent experiments performed in duplicate.

Association assays were performed by incubating *E. aerogenes*, *E. coli*, and *A. hydrophila* with *A. castellanii*. The non-associated bacteria were removed by washing amoebae in PBS for 3 times. The supernatant of the final wash was plated on nutrient agar plates but did not reveal any bacterial presence. Amoebae were then lysed using 0.5% SDS and lysates plated on nutrient agar plates. The findings revealed that *E. aerogenes* and *E. coli* demonstrated significantly reduced association with *A. castellanii* (1.99 ± 0.5 and 2.17 ± 0.8 bacteria:amoeba ratio respectively) as compared to *A. hydrophila* (7 ± 1.3 bacteria:amoeba ratio) (*P* < 0.01; using paired T-test, one-tail distribution) (Figure 1A). Here, the term association represents bacteria both inside amoebae and those that were attached on the surface of *A. castellanii*. A higher recovery of *A. hydrophila* (10.36% ± 1.2) of the original inoculum was observed as compared to *E. aerogenes* (2.44% ± 0.08) and *E. coli* (8% ± 0.9) (Figure 1B). Bacteria alone were incubated with various concentrations of SDS, and it was found that 0.5% SDS had no effect on bacterial viability (data not shown).

**Figure 2:**
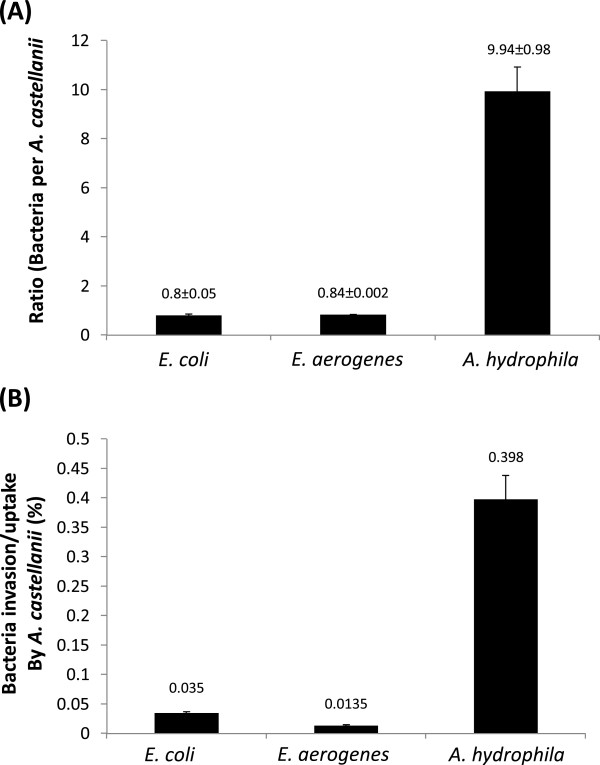
***Enterobacter aerogenes *and *Aeromonas hydrophila *that invaded and/or were taken up by *Acanthamoeba castellanii*.** Assays were performed to determine bacterial invasion into or phagocytosis by *A. castellanii* as described in “Methods”. (**A**) represents ratio of bacteria per amoeba, while (**B**) represents bacteria associated with amoeba (% of the original inoculum). Results are represented as the mean ± standard error of three independent experiments performed in duplicate.

Next, to determine the number of intracellular bacteria, invasion/uptake assays were performed. As per association, *A. hydrophila* exhibited higher invasion/uptake by *A. castellanii* (9.94 ± 0.98 bacteria:amoeba ratio) compared with *E. aerogenes* (0.84 ± 0.002 bacteria:amoeba ratio) (*P* < 0.01), or *E. coli* (0.8 ± 0.05 bacteria:amoeba ratio) (*P* < 0.01) (Figure 2A). A higher recovery of *A. hydrophila* (0.4% ± 0.002) of the original inoculum was observed (Figure 2B). Of note, the PBS post-gentamicin wash, plated onto nutrient agar plates did not yield any bacterial cfu, confirming that the antibiotic treatment was effective.

### *E. aerogenes, E. coli and A. hydrophila* survived intracellularly of *A. castellanii*

**Figure 3:**
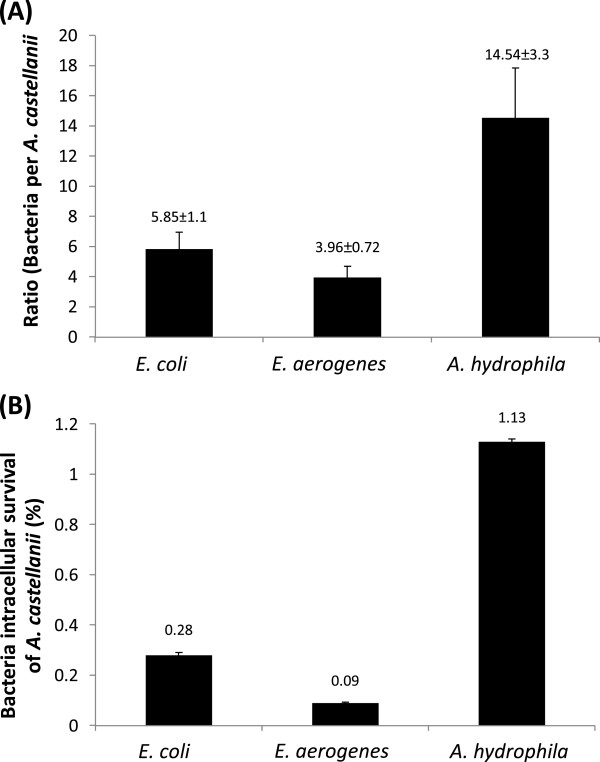
***Enterobacter aerogenes *and *Aeromonas hydrophila *that survived intracellularly from *Acanthamoeba castellanii *trophozoites.** Assays were performed to determine bacterial survival inside *A. castellanii* as described in “Methods”. (**A**) represents ratio of bacteria per amoeba, while (**B**) represents bacteria associated with amoeba (% of the original inoculum). Results are represented as the mean ± standard error of three independent experiments performed in duplicate.

Assays were performed to determine long term intracellular survival of *A. hydrophila, E. coli* and *E. aerogenes* inside *A. castellanii*. The findings revealed that all three bacterial isolates tested remained viable inside *A. castellanii*, while amoeba remained intact; however *A. hydrophila* exhibited higher survival inside amoebae (14.54 ± 3.3 bacteria:amoeba ratio), compared with *E. aerogenes* (3.96 ± 0.7 bacteria:amoeba ratio) (*P* < 0.05), or *E. coli* (5.85 ± 1.1 bacteria:amoeba ratio) (*P* < 0.05) (Figure 3A). *A. hydrophila* exhibited a higher recovery (1.13% ± 0.01) compared with *E. aerogenes* or *E. coli* (*P* < 0.05) (Figure 3B).

### Intracellular *E. aerogenes*, *E. coli* and *A. hydrophila* survived *A. castellanii* encystment process and were recovered from mature cysts

**Figure 4:**
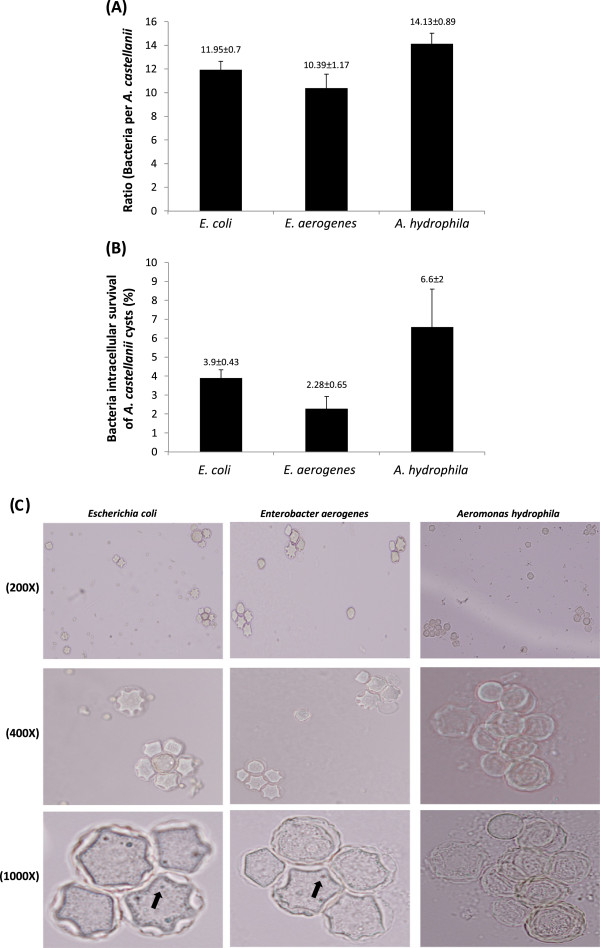
***Enterobacter aerogenes *and *Aeromonas hydrophila *encystment process of *Acanthamoeba castellanii *and recovery from mature cysts.** Assays were performed to determine bacterial survival inside *A. castellanii* during encystment and their recovery from mature cysts as described in “Methods”. (**A**) represents ratio of bacteria per amoeba, while (**B**) represents bacteria associated with amoeba (% of the original inoculum). Results are represented as the mean ± standard error of three independent experiments performed in duplicate. (**C**) representative micrograph of *A. castellanii* cysts containing *Escehrichia coli* K1, *Enterobacter aerogenes* and *Aeromonas hydrophila*. Images were taken using light microscopy under different magnifications (200×, 400×, 1000×). Arrows indicate bacteria.

Viability of *E. aerogenes, E. coli and A. hydrophila* inside *A. castellanii* during encystment and recovery from mature cysts was determined using intracellular cyst survival assays. The SDS treatment affected cyst viability (no growth observed in PYG) through lysing ostiole membrane leading to recovery of intracellular bacteria. The results showed that all three bacterial isolates tested remained viable during encystment (Figure 4). Interestingly, *A. hydrophila, E. coli* and *E. aerogenes* exhibited higher levels of recovery from mature cysts (14.13 ± 0.89 *A. hydrophila*:amoeba ratio, 10.3 ± 1.17 *E. aerogenes*:amoeba ratio, and 11.95 ± 0.7 *E. coli*:amoeba ratio) (Figure 4). Of the original inoculum, the bacterial recovery was 6.6 ± 2% for *A. hydrophila*, 2.28% ± 0.65 for *E. aerogenes* and 3.9 ± 0.43% for *E. coli* (*P* < 0.05).

**Figure 5:**
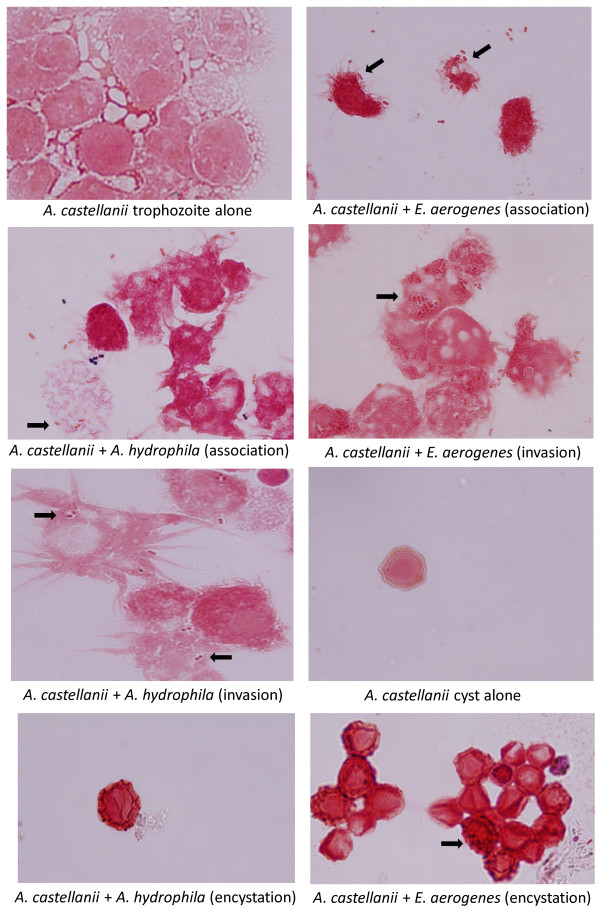
**Representative micrograph of *Acanthamoeba castellanii *interactions with *Escehrichia coli *K1, *Enterobacter aerogenes *and *Aeromonas hydrophila*.** Assays were performed to determine bacterial association with and invasion of *A. castellanii*, as well as bacterial survival inside *A. castellanii* cysts as described in “Methods”. Finally, samples were stained using Gram staining and observed under a microscope (×400). Note that extracellular bacteria are observed in association assays only, suggesting that gentamicin is effective in killing extracellular bacteria in both invasion as well as intracellular survival assays. Arrows indicate bacteria.

Additionally, co-cultures of *A. castellanii* trophozoites/cysts and *E. coli*, *E. aerogenes* and *A. hydrophila* were stained using Gram staining and observed under a microscope (×400). Note that extracellular bacteria are observed in association assays only, suggesting that gentamicin is effective in killing extracellular bacteria in both invasion as well as intracellular survival assays (Figure 5).

## Discussion

*Acanthamoeba* serve as a dock for various bacterial pathogens, e.g., *Legionella pneumophila* (the causative agent of Legionnaires’ disease), *Coxiella burnetii* (Q fever), *Pseudomonas aeruginosa* (keratitis), *Vibrio cholerae* (cholera), *Helicobacter pylori* (gastric ulcers), *Listeria monocytogenes* (listeriosis), *E. coli* and *Mycobacterium avium* (respiratory infections), and may act as a vector to transmit these pathogens to susceptible hosts, but the underlying mechanisms remain incompletely understood [19-21]. Here for the first time, the findings revealed that *A. hydrophila* and *E. aerogenes* associate with and survive inside *A. castellanii* trophozoites and cysts.

Notably, *A. hydrophila* exhibited higher association, invasion/uptake, and survival inside *A. castellanii* trophozoites compared with *E. aerogenes*. Both *A. hydrophila* and *E. aerogenes* remained viable during the encystment process and multiplied. These findings are consistent with previous studies, which showed that *A. castellanii* promoted the survival and growth of *C. jejuni*[22,23], *E. coli* K1 [18], and *L. pneumophila*[24,25]. However, as *Acanthamoeba* feeds on bacteria by phagocytosis, it is tempting to speculate that amoeba and bacteria are involved in convoluted interactions, the outcome of which is dependent on the virulence of bacteria [26]. For example, the non-invasive bacteria are taken up by *Acanthamoeba* as a food source, while the invasive bacteria are able to reside and possibly multiply inside amoebae without being killed [18], and use amoebae as (i) a transmission vehicle, (ii) training ground to develop resistance against other phagocytic cells such as human macrophages, and/or enhance virulence through evolution [27,28]. This is further strengthened with the fact that *Acanthamoeba* resemble human macrophages in many ways, particularly in their phagocytic activity and their interactions with various bacterial pathogens [28,29].

Encystment in *Acanthamoeba* is a complex process that involves morphological changes, termination of cell growth, removal of unnecessary materials, and results in the synthesis of at least two products not detected in trophozoites; cellulose [30] and an acid-insoluble protein-containing material [31]. In simple terms, the trophozoite becomes metabolically inactive (minimal metabolic activity) and encloses itself within a resistant shell. During the encystment process, RNA, proteins, triacylglycerides and glycogen levels are significantly decreased which results in decreased cellular volume and dry weight [32]. Keeping in mind these changes, we determined whether *E. aerogenes* and *A. hydrophila* can remain viable during the encystment process and be recovered from mature cysts or seen as excess materials and be discarded. Our results clearly demonstrated that both *A. hydrophila* and *E. aerogenes* survived the process of encystment and have the potential to exploit amoebae cysts as biological vectors. Although the molecular mechanisms of (i) precise localization of bacteria within amoebae using TEM, (ii) bacterial survival intracellular of amoebae trophozoites by evading phago-lysosome, (iii) inability of amoeba to dispose of bacteria during encystment, and (iv) how bacteria are retained within mature cysts are unclear, recent studies showed the presence of a diffusible factor produced by amoebae mediating survival and replication of *B. cepacia and V. parahaemolyticus*[33,34], which may explain our findings. In conclusion, the present study showed that both *A. hydrophila* and *E. aerogenes* exhibited association, invasion/uptake, and intracellular survival of *A. castellanii*, albeit *A. hydrophila* exhibited higher interactions compared with *E. aerogenes*. Therefore, *A. hydrophila* and *E. aerogenes* also joined the ranks of other bacteria that could benefit from *A. castellanii*.

## Conclusions

For the first time, it is shown that *A. hydrophila* and *E. aerogenes* associate with and survive intracellular of *A. castellanii* trophozoites and cysts. Thus, *A. hydrophila* and *E. aerogenes* also joined the ranks of other bacteria that could benefit from *A. castellanii*. Because cysts can be airborne, these findings suggest that *Acanthamoeba* is a potential vector in the transmission of *A. hydrophila* and *E. aerogenes* to susceptible hosts.

## Competing interests

The authors declare that they have no competing interests.

## Authors’ contributions

NAK conceived the study. FAY and RS designed and conducted all experiments under the supervision of NAK. FAY, RS, and NAK contributed to the writing of the manuscript. All authors approved the final manuscript.
